# Animal-Assisted Interventions for Neurodivergent Individuals: Advancing Research, Practice, and Well-Being

**DOI:** 10.3390/bs16050820

**Published:** 2026-05-19

**Authors:** Niko Kargas, Ana Maria Barcelos, Stamatina Tsiora

**Affiliations:** 1 Department of Cognitive Sciences, United Arab Emirates University, Al Ain P.O. Box 15551, United Arab Emirates; 2 School of Natural Sciences, University of Lincoln, Lincoln LN6 7TS, UK; abarcelos@lincoln.ac.uk; 3 School of Psychology, Sport Science and Wellbeing, University of Lincoln, Lincoln LN6 7TS, UK; mtsiora@lincoln.ac.uk

## 1. Introduction

Within contemporary clinical, educational, and psychosocial research, animal-assisted interventions (AAIs) occupy a distinctive and empirically contested position. Enthusiasm for the field has grown rapidly, driven by accounts of improved regulation, social engagement, confidence, and well-being across diverse populations. Yet the evidence base remains uneven in its terminology, methods, outcome frameworks, and explanatory models, and implementation in practice has frequently advanced ahead of the conceptual and methodological precision needed to support cumulative science (Beetz et al., 2012; Dimolareva & Dunn, 2021; Gee et al., 2021; Green et al., 2024; Kerulo et al., 2020; O’Haire, 2013, 2017; Rodriguez et al., 2021; Santaniello et al., 2020). This tension is especially visible in research involving neurodivergent populations, where interest in humane, relational, and non-stigmatising forms of support is high, and where outcomes, mechanisms, acceptability, and access cannot be assumed. Neurodevelopmental conditions are heterogeneous by definition (Thapar et al., 2017), and the neurodiversity framework usefully reminds us that such heterogeneity is not reducible to deficit alone but reflects variation in minds and nervous systems situated within social and environmental contexts (Chapman, 2020; Dwyer, 2022). This framing is particularly relevant here because, while autism and Attention-Deficit/Hyperactivity Disorder (ADHD) are paradigmatic neurodevelopmental forms of neurodivergence, the inclusion of Post-Traumatic Stress Disorder (PTSD) in this Reprint reflects an intentionally expansive and pragmatic use of the term. The scope of neurodivergence remains contested; however, trauma research demonstrates that PTSD is associated with enduring alterations in brain structure and function, alongside marked difficulties in emotion regulation and social functioning, domains that overlap meaningfully with those examined across autism and ADHD scholarship (Beheshti et al., 2020; Conti et al., 2023; McDonald et al., 2024; Pitman et al., 2012; Scoglio et al., 2022). Similarly, “animal-assisted intervention” is an umbrella term rather than a single modality, and the mere presence of an animal is not, in itself, a mechanism of therapeutic benefit (Kerulo et al., 2020; Santaniello et al., 2020). The central scholarly value of this Reprint lies precisely in how clearly it confronts that complexity.

## 2. Scope of the Special Issue

The ten contributions assembled in this Reprint comprise nine empirical studies and one systematic review. Across dogs, cats, and horses; across home, school, therapeutic, clinical, and community settings; and across autistic children, autistic adults, individuals with ADHD or co-occurring autism and ADHD, and veterans with PTSD, the Reprint moves beyond the simplistic question of whether animals “help” and towards more analytically productive questions: what kinds of human–animal relationships matter, for whom, in which contexts, by what processes, and under what practical, ethical, and methodological conditions (Browne et al., 2025; Brumpton & Kargas, 2026; Dimolareva et al., 2026; González-Sala et al., 2025; Hüsgen et al., 2025; Krause-Parello et al., 2025; Shoesmith et al., 2025; Toutain et al., 2025; Widdison et al., 2026; Wilson et al., 2025). Rather than serving as a simple catalogue of positive outcomes, the collection foregrounds important distinctions between intervention types, analytic levels, relational contexts, and forms of evidence, reflecting an important maturation in the field.

## 3. An Overview of the Published Articles

One of the clearest themes emerging across the collection is that the meaningful unit of analysis is not simply the animal, but the relational ecology in which human–animal contact is embedded. González-Sala et al.’s (2025) systematic review of 23 studies, conducted with PRISMA guidelines, anchors this point by showing that parents tend to perceive benefits of AAI at physical, social, and emotional levels, while also identifying family-level effects including reduced parental stress and improved family functioning. Yet this Reprint demonstrates consistently that such outcomes are shaped by far more than animal presence alone. Shoesmith et al. (2025), in a qualitative study of the Family Dog Service, found that parents described companion dogs as sources of enhanced attention, reduced loneliness and anxiety-based behaviour, and improved family well-being, but emphasised that these benefits were inseparable from tailored guidance, long-term support, and deliberate attention to sensory fit, dog selection, and relationship-building over time. Wilson et al. (2025) similarly showed that among autistic children, positive behaviour toward a pet predicted prosocial behaviour, while qualitative accounts highlighted emotion regulation, improved sleep, comfort, and expanded social opportunity alongside pet-related anxieties and welfare concerns. Widdison et al. (2026) extend this picture further by examining cat–human interaction in autistic and ADHD adults, showing that companion-animal relationships can also involve burden: anxious cat behaviour, poorer cat health, close proximity, and perceived inability to provide adequate care for the animal were each associated with poorer well-being outcomes. Taken together, these studies challenge the still-common assumption that pet ownership or animal contact is inherently salutogenic. The more defensible conclusion is that human–animal relationships can function as psychosocial resources, but only under specific relational, practical, and ethical conditions (Atherton et al., 2023; Barcelos et al., 2021; Hall et al., 2016; O’Haire, 2013).

A second major contribution of this Reprint is its attention to mechanism and process, addressing a recurring weakness in AAI scholarship: the tendency to report outcomes without adequately explaining how they arise (Nieforth et al., 2021; Rodriguez et al., 2021). Several papers directly confront this gap. Hüsgen et al. (2025), using behavioural observation and an ethogram across repeated dog-assisted therapy sessions, found partial support for the “icebreaker” account: participant–dog interaction tended to decrease over time while participant–therapist engagement increased, suggesting that the dog may initially facilitate rapport rather than remaining the central relational focus throughout therapy. Toutain et al. (2025) add a further layer of analytical sophistication by combining eye-tracking, ethological observation, and facial expression identification tasks. Their findings demonstrate that visual attention is context dependent: children diagnosed with autism paid less attention to faces and more attention to the broader environment than their typically developing peers, yet behavioural patterns during free interaction with an assistance dog were similar across groups, and within-group associations linked gaze allocation to interaction style and expression-recognition performance. Wilson et al. (2025) further bridge process and outcome by suggesting that prosocial development may be linked less to attachment in the abstract than to the quality of enacted behaviour toward the pet. Across these papers, animals are not treated as quasi-magical therapeutic agents but as participants in dynamic, situated systems of attention, arousal, co-regulation, and relational learning, a position that is consistent with broader biopsychosocial accounts of human–animal interaction (Beetz et al., 2012; Gee et al., 2021; O’Haire et al., 2015).

The equine-focused contributions press the field towards greater precision in intervention design. Browne et al. (2025) examine the acceptability of Occupational Therapy Using Zones of Regulation^TM^ concepts in an equine environment for autistic children, making a particularly valuable methodological and ethical contribution by centring the perspectives of the children themselves. Their findings are instructive: the intervention was generally acceptable, horse riding was clearly meaningful and enjoyable, yet some components of the Zones of Regulation^TM^ curriculum were experienced as less acceptable, illustrating that promising interventions may still require adaptation in delivery, pacing, or content to align with participant preferences and capacities. Additionally, Brumpton and Kargas (2026) conducted a qualitative study of therapeutic horse riding for autistic adults, showcasing positive outcomes such as enjoyment, calm, self-efficacy, authenticity in relationships, and benefits that extended into daily life beyond the riding arena, while also highlighting the structural barriers (i.e., cost, transport, limited facilities, limited specialist staff) that determine who can actually participate. Together, these papers support the broader equine-assisted services literature while pressing it in a more differentiated direction: away from generic claims about horses as therapeutic and toward a more empirically grounded account of equine-assisted services as embodied, relational, structured, and access-dependent forms of support (Gabriels et al., 2015; McDaniel Peters & Wood, 2017; Wood et al., 2021).

This Reprint is equally notable for situating the benefits of canine-assisted interventions in different settings. Dimolareva et al. (2026) analysed handler and teacher reports from 58 schools participating in The Dog Mentor programme, showing how school-based dog support is perceived to create calmer environments, promote engagement, reduce barriers to learning, and support self- and emotion regulation. Importantly, this study highlights that flexibility may not be merely a methodological concession but a feature that makes school-based AAI workable in practice. Shoesmith et al.’s (2025) findings indicated that companion dogs could have similar benefits to those reported for assistance dogs for autistic children, but also emphasised the importance of practical scaffolding in decision making with respect to selecting and training a dog. Krause-Parello et al.’s (2025) randomised study of female veterans with PTSD participating in a service dog training programme highlighted its positive effects on a number of biological and psychological indicators of stress, whereas combat exposure moderated these changes. This is a valuable reminder that the effects of animal-assisted programmes may be multidimensional, non-linear, and context sensitive.

## 4. Methodological and Conceptual Considerations

Methodologically, this Reprint is stronger than much of the preceding literature. Its contributions include a PRISMA-guided systematic review with a registered protocol, qualitative interview studies with parents and autistic adults, mixed-methods research, content analysis, observational coding of therapeutic triads, eye-tracking, and biomarker assessment. This diversity is a genuine strength, and it also exposes a number of limitations. Samples are often small; participant groups are sometimes internally heterogeneous; direct participant voice remains less common than proxy report; and outcome frameworks remain difficult to compare across studies. These challenges are not unique to AAI research, but they are particularly consequential in a field characterised by substantial variability in species, training, setting, handler expertise, participant preference, and programme aims (O’Haire, 2017; Rodriguez et al., 2021). What emerges from the Reprint is not a call for rigid standardisation at the expense of person-centred practice, but for sharper and more transparent reporting, clearer conceptual boundaries, and more explicit theory of change. The field needs frameworks that can accommodate both benefit and burden, symptom change and positive well-being, and human outcomes and animal welfare. Widdison et al.’s (2026) use of a pet–human-related factors framework is instructive in this respect, capturing both the positive as well as cumbersome aspects of cat ownership rather than assuming that closeness is uniformly beneficial (see also Barcelos et al., 2021).

A further scholarly strength of this Reprint is its alignment with neurodiversity-affirming priorities, even where the underlying literature reflects mixed terminology and divergent conceptual traditions. Several papers explicitly treat lived experience as a primary source of knowledge rather than a supplement to clinician- or caregiver-generated data. Browne et al. (2025) centre autistic children’s accounts of intervention acceptability. Brumpton and Kargas (2026) foreground autistic adults’ own accounts of meaning, access, and psychological change. Wilson et al. (2025) and Shoesmith et al. (2025) take family perspectives seriously without reducing children’s experiences to parental interpretation. This represents an important corrective in a field that has historically spoken about neurodivergent people more readily than with them. More broadly, the Reprint models a shift in autism and neurodiversity research: from deficit-framed, externally defined notions of “improvement” towards more situated questions about fit, safety, communication, agency, and quality of life (Atherton et al., 2023; Botha & Cage, 2022). At the same time, the inclusion of veterans with PTSD within the Reprint’s scope invites productive conceptual reflection. It signals an intentionally expansive understanding of neurodivergence organised less by diagnostic purity and more by shared concerns around regulation, relational safety, mental health, and access to supportive environments. Whether all such conditions are best grouped under a common conceptual umbrella remains open to debate, but the Reprint demonstrates that this boundary question can be intellectually productive rather than merely terminological.

## 5. Practical Implications and Future Directions

The practical implications of this body of work are substantial. For clinicians, this Reprint suggests that animal-assisted work should be treated neither as an all-purpose adjunct nor as a niche novelty, but as a set of structured relational options whose appropriateness depends on the person, animal, context, goals, and available support infrastructure. For educators and school leaders, the school-based studies point to the importance of staff training, whole-setting planning, and animal welfare safeguards rather than ad hoc or unstructured use. For service providers, the collection foregrounds access and equity as central rather than secondary concerns: cost, transport, waiting times, staffing, and local availability all determine who benefits from promising programmes. For researchers, the Reprint underscores the need for comparative work across intervention types, longitudinal follow-up, more precise moderator analyses, economic and implementation evaluation, and co-produced designs that take neurodivergent priorities seriously from the outset. Such priorities are not incidental to scientific progress; they are necessary if the field is to move from pockets of encouraging evidence to genuinely evidence-based, ethically defensible, and scalable practice.

## 6. Conclusions

What, finally, does this Reprint contribute to the wider field? Its greatest contribution may be that it resists simplification. It does not present AAIs as universally effective, inherently benign, or conceptually settled, nor does it dismiss the field on account of its methodological heterogeneity. Instead, it shows a field in the midst of methodological and theoretical consolidation. Animals in these studies appear as companions, co-regulators, catalysts for social contact, partners in embodied activity, and, at times, sources of obligation or strain. Neurodivergent participants are not treated as passive recipients of benefit, but as individuals whose sensory profiles, communicative styles, preferences, histories, and material circumstances shape what any intervention can become. In this sense, the Reprint advances research, practice, and well-being not by closing debate, but by improving its quality: making it more precise, more critical, more ethically alert, and more responsive to lived experience.

## Abbreviations

The following abbreviations are used in this manuscript:

AAIs	Animal-Assisted Interventions
ADHD	Attention-Deficit/Hyperactivity Disorder
PTSD	Post-Traumatic Stress Disorder
